# Comparative analysis of endophytic bacterial localization and microbiome diversity in plant varieties under varied growth conditions through microscopic imaging and sequencing techniques

**DOI:** 10.3389/fmicb.2025.1568209

**Published:** 2025-05-16

**Authors:** Mohd Shadab, Suman Kumar Samanta, Jilmil Baruah, Sujata Deka, Garima Raj, Narayan C. Talukdar

**Affiliations:** ^1^Faculty of Science, Assam down town University, Guwahati, Assam, India; ^2^Life Sciences Division, Institute of Advanced Study in Science and Technology, Guwahati, Assam, India

**Keywords:** endophytic bacteria, intracellular colonization, microbiome diversity, next-generation sequencing, plant-microbe interactions

## Abstract

**Introduction:**

Intracellular colonization by endophytic bacteria (EB) is a relatively new and less explored aspect of plant microbiome research. In this study, we investigated the presence and localization of EB in *Nicotiana tabacum* var. Podali and *Vigna radiata* var. Pratap using SYTO9 (S9) and Propidium Iodide (PI) staining.

**Methodology:**

Confocal Laser Scanning Microscopy (CLSM) was used to visualized bacterial localization, MitoTracker Deep Red (MDR) was used to confirm non- overlapping mitochondrial staining. Time-lapse imaging was employed to observe bacterial motility. For microbial community profiling next-generation sequencing (NGS) of the 16S rRNA gene was conducted to analyze bacterial diversity and composition.

**Results:**

Confocal laser scanning microscopy (CLSM) revealed S9-labelled live bacteria located close to the nucleus in Podali tissues and suspension cultures, while PI selectively stained dead cells. MitoTracker Deep Red (MDR) confirmed that there was no overlap with mitochondrial staining. Interestingly, time-lapse imaging captured the movement of bacteria within the cells, indicating possible bacterial motility. EB were observed in both in vitro and field-grown Podali plants, whereas they were detected only in field-grown Pratap plants. Next-generation sequencing revealed that Podali harbored a much higher bacterial diversity, with 37 bacterial families identified mainly from *Burkholderiaceae* and *Enterobacteriaceae*. In contrast, Pratap plants showed lower diversity, with only 10 bacterial families, dominated by *Rhizobiaceae*.

**Conclusion:**

This study is among the first to report intracellular EB localization in these plant varieties and demonstrates how environmental conditions and growth methods can influence the composition of plant-associated microbiomes.

## 1 Introduction

Endophytic bacteria (EB), are non-pathogenic microorganisms that reside in plant tissue without causing harm (Kandel et al., 2017). These include bacteria and fungi that form intricate association with plants, contributing to various physiological processes, although their exact functions remain incompletely understood (Jia et al., 2016). These can be categorized as facultative, obligate, or passenger, depending on their life cycle and role in host plan (Hardoim et al., 2008). EB typically enter through roots or shoots and colonize internal niches such as intercellular spaces, vascular bundles, or, in rarer cases, intracellular compartments (Frank et al., 2017; Kandel et al., 2017). While intercellular colonization is well documented, intracellular colonization remains a relatively unexplored area of research.

Previous studies using Transmission Electron Microscopy (TEM) suggest bacterial transmission from the cytosol into cell vacuoles (de Almeida et al., 2009). TEM and CLSM studies have revealed that bacteria associate internally with their hosts, both intercellularly and intracellularly, in fungal hyphae and recently in plant tissue (Bianciotto et al., 2004; Bianciotto et al., 2003; Thomas and Franco, 2021). With the advancement of metagenomic techniques, diverse bacterial and fungal communities have been comprehensively documented in plants (Ramakrishnan et al., 2024). However, limitations in understanding nutrient requirements hinder the isolation of all bacteria using culturable methods (Zhang et al., 2024).

Several visualization techniques, such as fluorescence-based tagging and viability staining, have been employed to monitor endophytic colonization and movement within plant tissues (Thomas and Franco, 2021; Thompson and Raizada, 2024; Posada et al., 2018).

SYTO9 (S9) and Propidium Iodide (PI) are widely used to differentiate between live and dead bacteria in various biological samples based on membrane integrity (Verma et al., 2021; Rosenberg et al., 2019). However, the specificity of stains like S9 in differentiating bacterial DNA from organelle DNA remains unclear (Chávez-González et al., 2024).

In this study, *Vigna radiata* var. Pratap and *Nicotiana tabacum* var. Podali were chosen due to their traditional medicinal relevance and bioactive compounds (Kabré et al., 2022; Zhang et al., 2024).

The research aimed to localize EB in aseptically grown tissues and cell suspension cultures and to evaluate whether SYTO9 can selectively stain bacterial cells without cross-reacting with plant organelles.

## 2 Materials and methods

### 2.1 Seeds surface sterilization

*Nicotiana tabacum* var. Podali was sourced from the Central Tobacco Research Institute in Dinhata, West Bengal, India, while *Vigna radiata* (green gram) var. Pratap was obtained from Krishi Vigyan Kendra, Shilongoni, Assam, India. Seed surface sterilization for both Podali and Pratap followed the protocol outlined by Hardoim et al. (2012) with slight modifications. For Podali seeds, the sterilization process involved several steps: First, the seeds were treated with 70% ethanol for 30–45 s. This was followed by treatment with 10% hydrogen peroxide (H₂O₂) for 20 min in a shaking incubator set at 30°C and 200 rpm. The seeds were then thoroughly rinsed with sterilized water. Next, the seeds were exposed to a sterilization solution consisting of 0.4% sodium hypochlorite (NaOCl), 0.1% sodium carbonate, 3% sodium chloride, and 0.15% sodium hydroxide at 30°C for 20 min in a shaking incubator at 200 rpm. To eliminate any surface-adhered NaOCl, the seeds were washed 5–6 times with 2% sodium thiosulfate at 30°C for 10 min at 200 rpm. To ensure sterility, the surface-sterilized seeds were imprinted on nutrient agar (NA) plates and placed in nutrient broth (NB) tubes. The plates and tubes were incubated at 28°C in a BOD incubator and a shaking incubator for 5 days, respectively.

For Pratap seeds, a similar surface sterilization protocol was followed. However, after the 70% ethanol treatment, the seeds were treated with 0.2% sodium hypochlorite for 20 min, and similarly washed with 2% sodium thiosulfate, omitting the hydrogen peroxide (H₂O₂) treatment.

### 2.2 Callus induction and suspension culture

Surface sterilized Podali seeds were germinated on a 0.7% agar medium. After 5 days, the seedlings were transferred to Murashige and Skoog (MS) basal medium (Maher et al., 2020), supplemented with 30 g/L sucrose and solidified with 0.7% agar. Following a 10–15-day incubation period, 50 plants were selected for callus induction. Root and shoot explants were excised and cut into small segments using a sterile razor blade. To optimize callus formation, the explants were cultured on MS medium containing various combinations of the auxins 2,4-dichlorophenoxyacetic acid (2,4-D) and 1-naphthaleneacetic acid (NAA), along with the cytokinin 6-benzylaminopurine (BAP). The pH of the medium was adjusted to 5.6–5.8. Callus induction was evaluated under both photoperiodic conditions (16 h light/8 h dark) and complete darkness. Cultures were maintained at 25 ± 2°C with 60% relative humidity and a light intensity of 67.50 μmol/m^−2^/s^−1^.

A cell suspension culture was initiated from friable callus obtained after 3–4 weeks. A single callus fragment (0.5–0.8 g) was inoculated into MS medium supplemented with 2 mg/L 2,4-D and 0.5 mg/L BAP. The pH was adjusted to 5.6–5.8 prior to autoclaving. The cultures were kept in the dark at 25 ± 1°C in a shaking incubator set at 100 rpm.

### 2.3 Fluorescent staining and microscopy analysis

LIVE/DEAD BacLight® bacterial viability kit L13152 (Molecular Probes, Thermo Fisher Scientific) containing SYTO-9 and propidium iodide (PI) was employed for detecting live/dead bacteria with the stocks prepared in autoclaved water.^1^ S9 binds to the nucleic acids (NuA) of both viable and non-viable bacterial cells, whereas PI selectively binds to dead or membrane-compromised cells. PI exhibits a higher binding affinity for NuA than S9, which can inhibit S9 binding to damaged cells (Rosenberg et al., 2019). Confocal laser scanning microscopy (CLSM) was used to visualize endophytes and organelles in Podali (leaf tissues and cells from cell suspensions), Pratap (leaf tissues), as well as bacteria and organelles in MCF-7 breast cancer cell, cardiac and skeletal muscle tissues from Swiss albino mice. Ten one-month-old Podali plants, six 20-day-old Pratap plants grown *in vitro*, five Pratap plants (ranging from 20 days to one month old) grown in open field conditions, and tissue samples from three Swiss albino mice were examined using CLSM. For each plant, four slides were prepared from individual leaf tissues and observed under CLSM. Cell suspensions were derived from a single callus, subculture in multiple flasks, and observed under CLSM, with the experiment repeated three times. All procedures were performed under aseptic conditions. 1 mL of cell suspension from one-month-old Podali plants was collected into a 1.5 mL centrifuge tube, and the medium was removed by aspiration. The remaining cell mass was washed with sterile distilled water and then incubated with a cocktail of 30 μM Propidium Iodide (PI) and 6 μM SYTO 9 (S9) for 20 min at room temperature to assess cell viability, membrane integrity, and bacterial contamination. To distinguish between bacteria and mitochondria in the cell suspensions, the cells were incubated with 400 nM MitoTracker Deep Red (MDR) for 45 min at 35 ± 2°C in the dark. The cells were then washed six to seven times with sterile distilled water and further incubated with S9 for 20 min at room temperature.

For CLSM imaging and staining of leaf tissues, free-hand sections of approximately 50–100 μm in thickness were prepared using a sterile razor blade and stained with 6 μM S9, following the same protocol as the cell suspensions. Chloroplast autofluorescence was also observed. To verify the specificity of S9 staining, cardiac and skeletal muscle tissues from Swiss albino mice were used as a negative control. Prior to sectioning, the tissues were washed with 70% ethanol for 1 min to remove any surface bacterial contamination. Thin, free-hand deep tissue sections were then prepared using a sterile razor blade (Bader et al., 2018). The staining protocol for the tissue sections and MCF-7 human cancer cell line, including incubation with MDR and S9, followed the same approach as for the cell suspensions. After staining, cancer cell and the tissue sections were examined under CLSM.

Cells were imaged using a 561 nm DPSS (diode-pumped solid-state) laser with emission at 617 nm after PI staining. MDR-stained tissues were imaged using a 633 nm HeNe laser with emission at 665 nm, while S9-stained samples were imaged using a 488 nm argon-ion laser with emission at 498 nm. The 488 nm argon laser was used to excite S9, and the 633 nm laser for MDR excitation. Chloroplast autofluorescence in green gram and tobacco leaf tissues was observed using a 488 nm argon-ion laser with emission between 680 and 700 nm.

### 2.4 Video and 3D construction

Z-stack image acquisition for 3D reconstruction was carried out using a Leica DMi8 confocal laser scanning microscope with a 63x oil-immersion objective (1.5 numerical aperture). Step sizes of 2–3 μm were applied based on the thickness of the samples. Three-dimensional image and video generation were performed using LAS X Leica software. Image labeling was completed in Microsoft PowerPoint, and manual object tracking was conducted with the ImageJ/Fiji plugin.

### 2.5 Next generation sequencing, data processing, and bioinformatics analysis

Total DNA was extracted from Podali and Pratap plants following the method described by Sharma et al. (2002). For Podali, a single plant shoot was collected per replicate, while for Pratap, 20 plant leaves were sampled for each replicate. Total 3 replicate of Podali (TS_1, TS_2 and TS_3) and 3 replicate of Pratap (PS_1, PS_2 and PS_3) were analyzed in this study. The V3-V4 hypervariable region of the 16S rRNA gene was selected for next-generation sequencing. Libraries were prepared to generate 2 × 300 paired-end reads on the Illumina MiSeq platform, with sequencing performed by Macrogen Inc., South Korea. Demultiplexing was carried out using the q2-demux tool in QIIME2, followed by denoising with the DADA2 algorithm within QIIME2, which generated amplicon sequence variants (ASVs). After applying quality filtering and removing chimeras, high-quality sequences were clustered into OTUs at a 97% similarity threshold. Read counts varied between the Podali and Pratap samples. All experiments were conducted under aseptic conditions. Relative and absolute abundance plots were created in R, retaining all OTUs, including those with low abundance, for diversity analysis after constructing the OTU table.

## 3 Results

### 3.1 Surface sterilization validated by absence of bacterial contaminants

After surface sterilization of the seeds, no bacterial growth was detected on Nutrient Broth (NB) or Nutrient Agar (NA) plates, indicating that surface contaminants were successfully removed. To further verify the sterilization process, Scanning Electron Microscopy (SEM) was used. The SEM images showed no bacterial colonies on the seed surfaces or the leaves of the germinated plants, confirming the effectiveness of the sterilization (Supplementary Figure S1). These findings ensure that the surface sterilization protocol was effective in eliminating external bacterial contamination, thereby ensuring the integrity of subsequent experiments.

### 3.2 Optimization of callus induction and cell suspension culture

Callus induction from shoot explants demonstrated variable responses depending on the plant species and hormonal combinations. Shoot explants from Podali did not exhibit successful callus formation, instead undergoing browning under both tested phytohormonal combinations—2,4-D (2 mg/L) and BAP (0.5 mg/L), as well as NAA (2 mg/L) and BAP (0.5 mg/L). This browning suggests a lack of responsiveness or possible tissue damage, highlighting the challenge of optimizing callus induction in this plant species.

In contrast, root explants exhibited 100% callus induction, particularly in the presence of 2 mg/L NAA and 0.5 mg/L BAP under light conditions as shown in Table 1. The generated callus was friable, a key indicator of its suitability for further cell suspension culture. Cell suspension cultures were initiated by transferring pieces of this friable callus to MS media supplemented with 2 mg/L 2,4-D and 0.5 mg/L BAP. Cell dispersion was observed within 3–5 days of culture initiation, with optimal dispersion achieved after 15–20 days. The friable nature of the callus and the timely dispersion into single cells demonstrated the success of the optimization process for cell suspension culture initiation.

**Table 1:** Effects of different phytohormone concentrations in MS basal medium on callus induction.

Sl. No.	Phytohormones	Concentration (mg/l)	Different organs	Light/dark	Nature of callus
1	2,4-D	2	Leaves	Dark	White/black
	BAP	0.5			
2	NAA	2	Leaves	Dark	White/black
	BAP	0.5			
3	2,4-D	2	Root	Dark	Brown and compact
	BAP	0.5			
4	NAA	2	Root	Dark	Whitish and compact
	BAP	0.5			
5	NAA	2	leaves	Light	Brown
	BAP	0.5			
6	NAA	2	Root	Light	Green compact/ friable
	BAP	0.5			

Callus induction was performed using root and shoot explants. The medium supplemented with different concentration of phytohormones in light and dark condition.

### 3.3 Mitochondrial and bacterial differentiation

**Figure 1:**
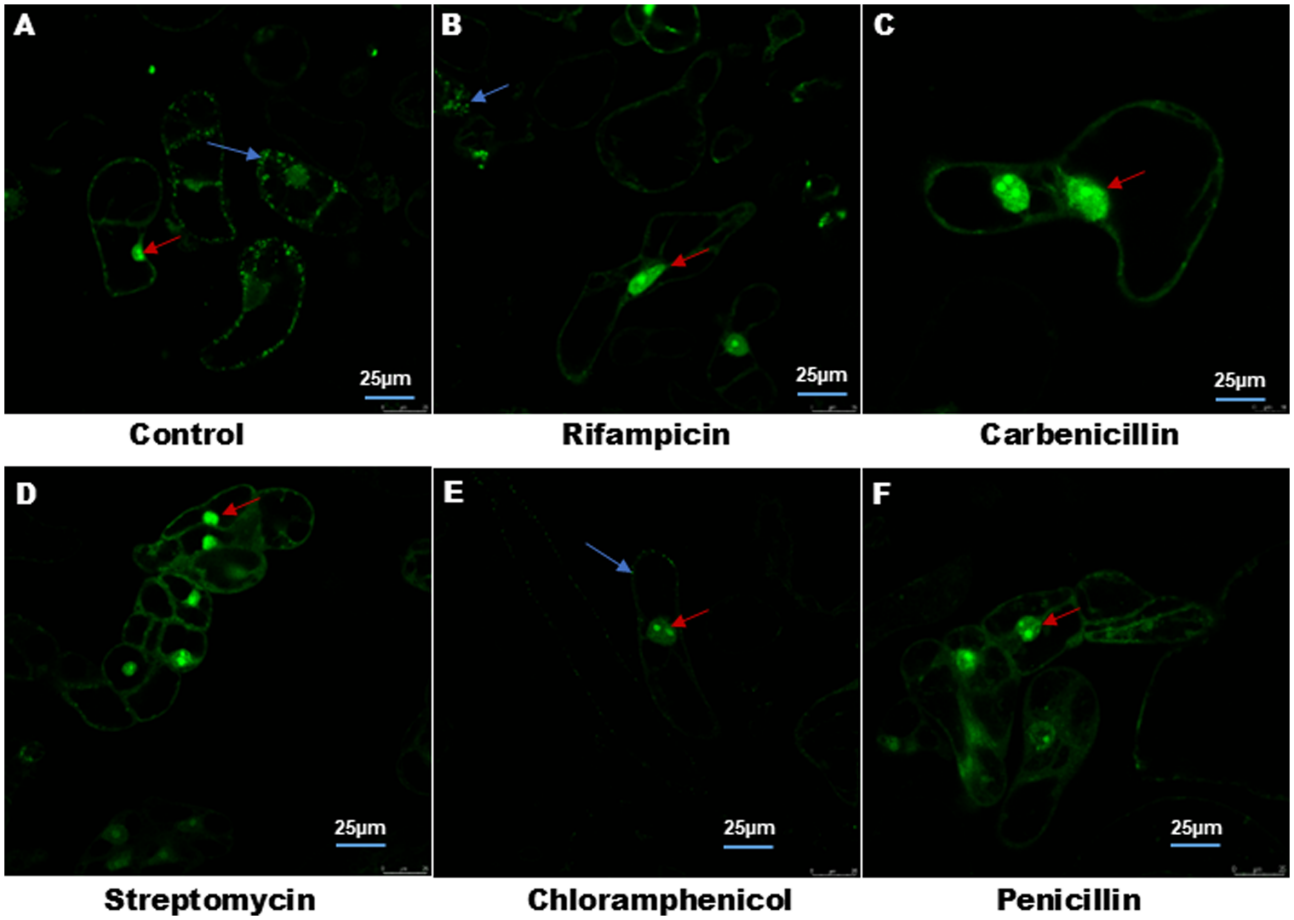
Treatment of plant cell suspension with different antibiotics. **(A)** Control plant cell suspension stained with Syto9 shows endophyte around the nucleus and cell boundary shown by blue arrow. **(B–F)** Plant cell suspension treated with different antibiotics (100 μg) showed elimination of endophyte. In **(B)** rifampicin treated cell suspension display few bacteria cells. In each cell, nucleus was stained with Syto9 shown with red arrow. Image size 25 μm.

**Figure 2:**
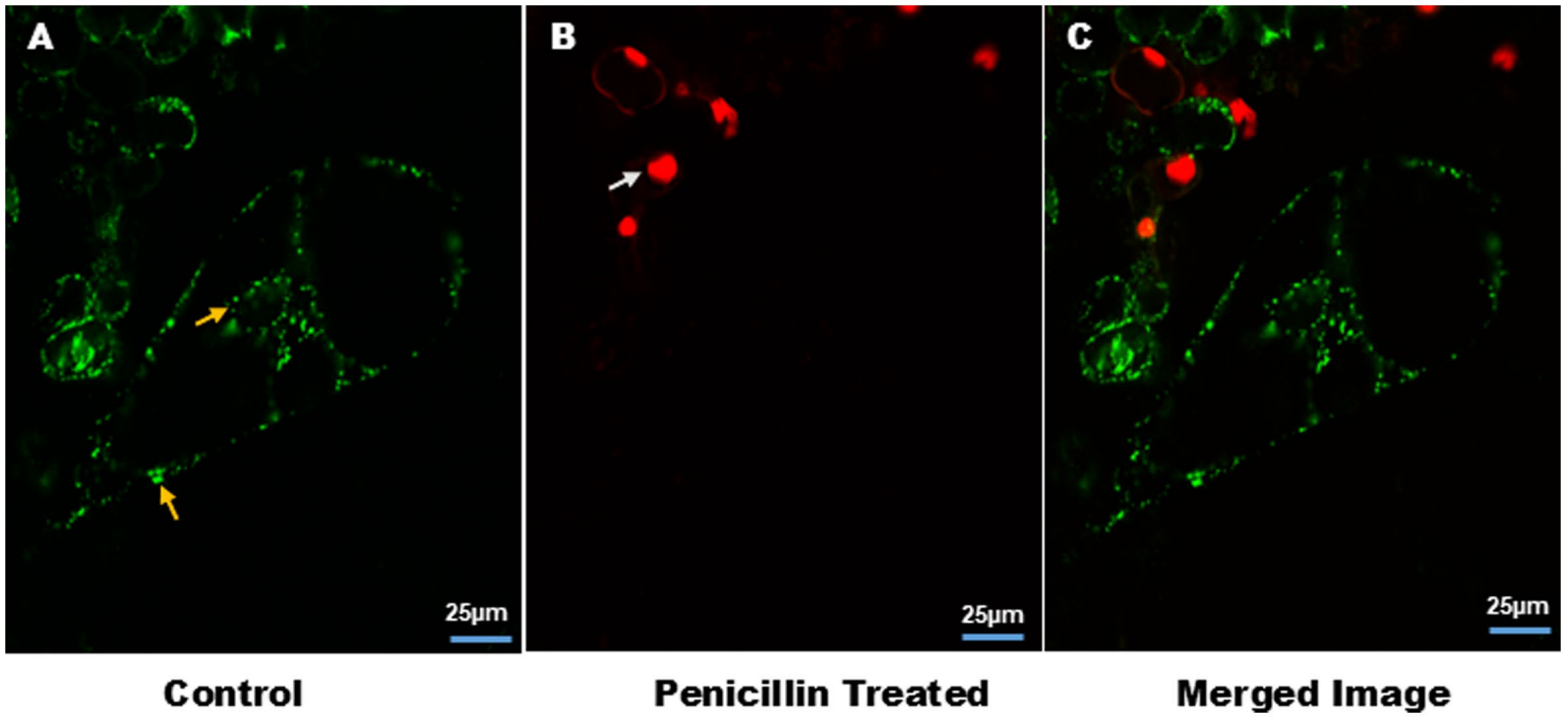
Cell viability in plant cell suspension and intracellular association of EB. **(A)** Yellow arrow showed staining of EB by S9 in single cell suspensions, around the nucleus and plasma membrane. **(B)** White arrow indicates staining of nucleus with PI of non-viable cells. **(C)** Merge image of **(A,B)**. Image size 25 μm.

Figure 1 shows the effect of different antibiotics on bacterial endophyte in plant cell suspension stained with S9. In control plant cell suspension, EB was observed within the cells, while in antibiotic-treated cells suspension, some bacteria were observed in cells treated with chloramphenicol and rifampicin. Figure 2 illustrates the selective staining properties of PI and S9. PI successfully stained only non-viable cells, specifically those treated with antibiotics, while S9 exclusively labeled viable cells. Notably, no bacterial signatures were detected in PI-positive (non-viable) cells, whereas S9-positive (viable) cells exhibited distinct intracellular bacterial presence, particularly in proximity to the nucleus. Importantly, no extracellular bacterial signatures were observed in either staining condition, confirming the absence of external contamination.

**Figure 3:**
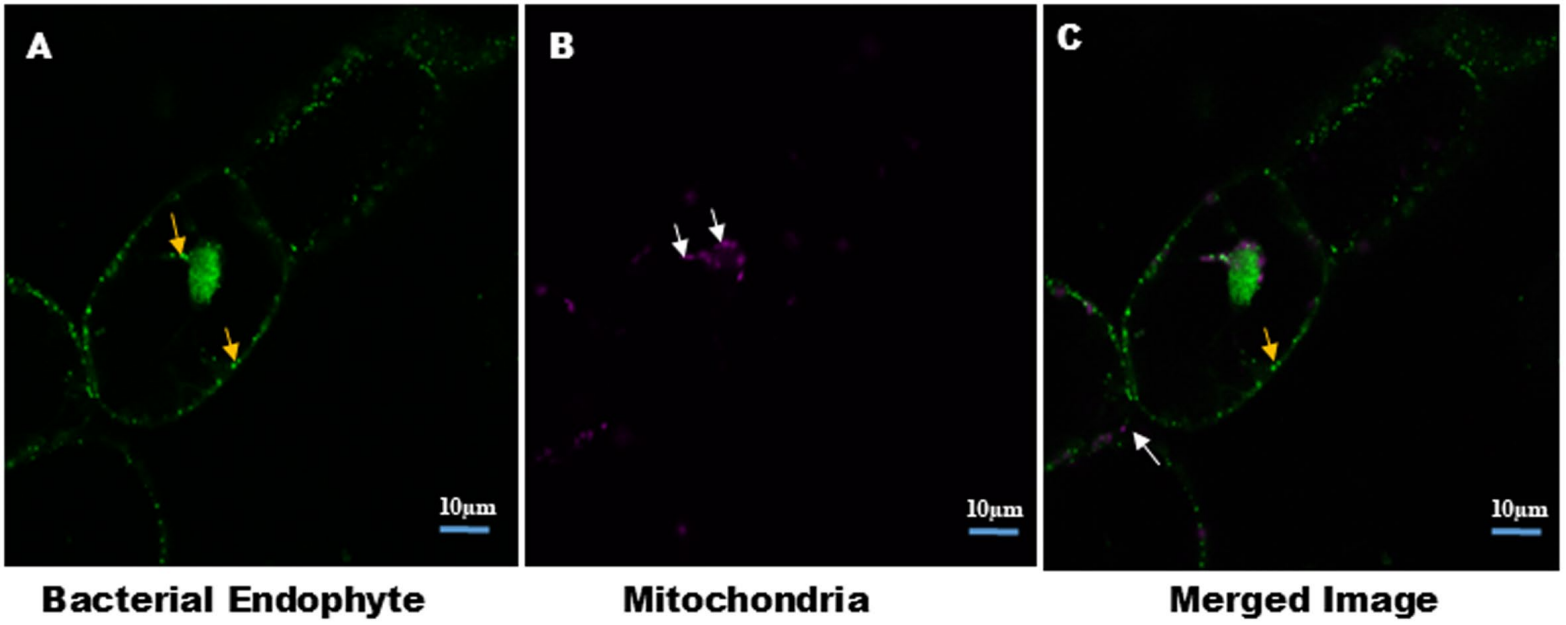
Distinction between intracellular bacteria and mitochondria in plant cell suspension culture. Cells were stained with MDR for mitochondria and S9 for bacteria and nucleus. Confocal micrograph show: **(A)** intracellular bacteria stained with S9, indicated by yellow arrow. **(B)** Mitochondria stained with MDR, indicated by white arrow. Size of the mitochondria ranges from 1.45–2.27 μm. Presence of bacteria and mitochondria together indicates the cytosolic nature of EB. Image size 10 μm.

Figure 3 further clarifies the distinction between bacterial and mitochondrial structures (Supplementary Video V7), with bacteria predominantly located near the nucleus and at the cell periphery. In this analysis, S9 stained both the nucleus and intracellular endophytic bacteria (EB) (Supplementary Figure S2), while the mitochondrial-specific dye (MDR) exclusively labeled mitochondria. The Size of mitochondria ranged from 1.45 to 2.27 μm, whereas bacteria measured between 0.67 and 1.2 μm, highlighting the morphological differences. Additionally, bacterial motility and chloroplast movement were observed within individual cells. This bacterial movement, as well as cytoplasmic streaming, was further visualized in suspension cells, as shown in time lapse video (Supplementary Videos V1, V2).

### 3.4 Endophyte visualization using confocal microscopy

**Figure 4:**
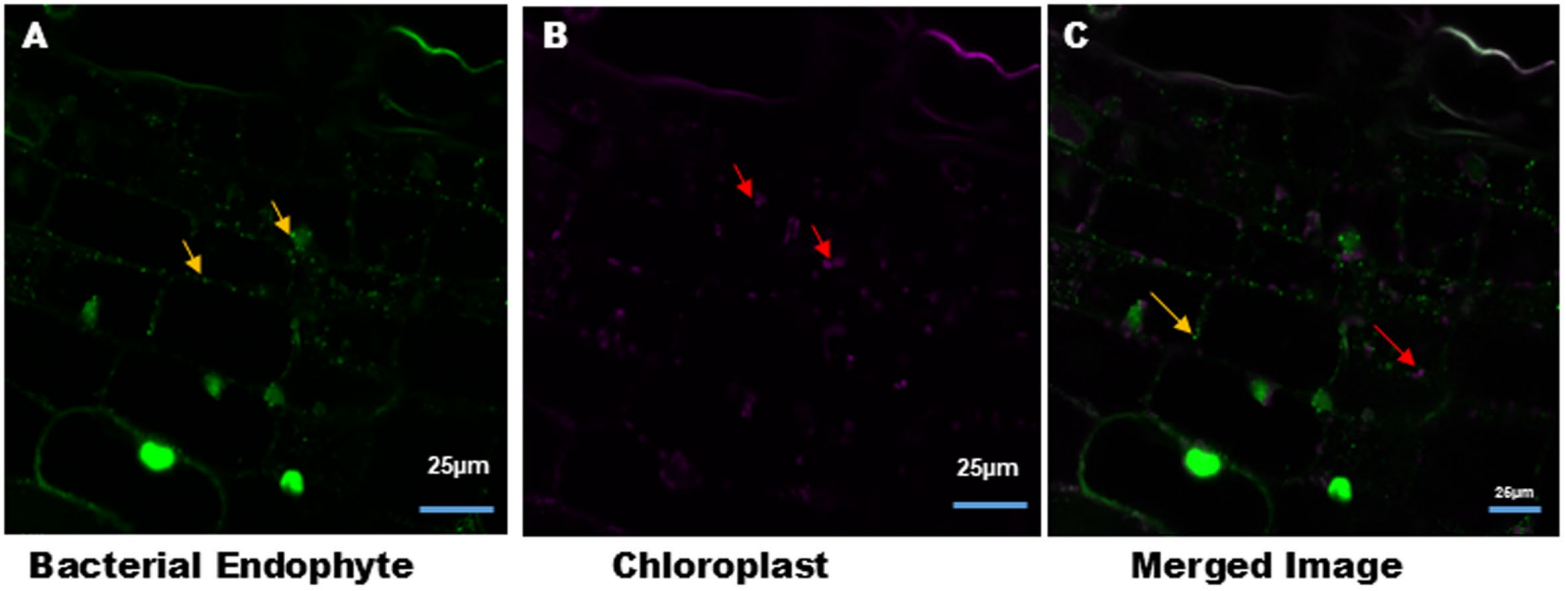
Intracellular bacteria and chloroplast in the tobacco leaves tissue. **(A)** Syto9 staining of tobacco leaves tissue section showed the presence of intracellular bacteria (green color) around the nucleus and the cell membrane, indicated by yellow arrow. **(B)** Auto-fluorescence of chloroplasts (magenta color) from leaves tissue indicated by red arrow. **(C)** Merged image of **(A,B)**. Image size 25 μm.

Endophytic bacteria were clearly observed in the leaf tissues of Podali plants grown under *in vitro* conditions. Confocal microscopy revealed the distinct auto-fluorescence of chloroplasts, which allowed for the clear differentiation between chloroplasts and bacteria in the Podali leaf tissue (Figure 4). With the cell suspension cultures, S9 selectively stained endophytic bacteria and the plant cell nucleus but did not stain chloroplasts.

**Figure 5:**
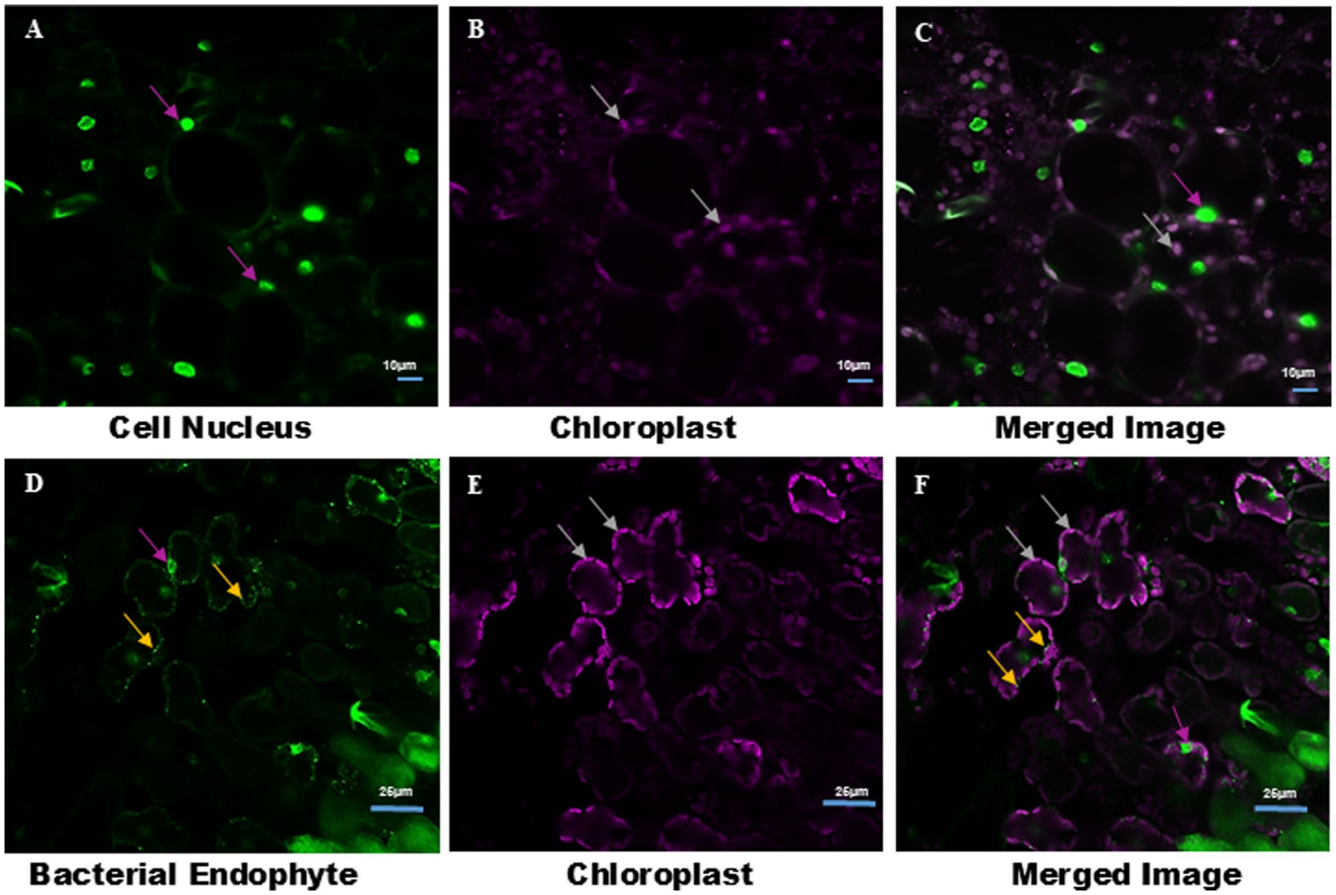
Observation of endophytic bacteria in Pratap Plants grown under aseptic condition and in open field using LCM. **(A)** S9 stains only the nucleus (indicated by magenta arrows) of leaf tissue of Pratap plants grown under aseptic condition in MS medium. **(B)** Auto-fluorescence of chloroplast in aseptically grown Pratap plants (indicated by white arrow). **(C)** Merge image of **(A,B)**. **(D)** S9 stains both the nucleus (indicated by magenta arrow) and endophytic bacteria (indicated by white arrow) in leaf tissue sections of Pratap plants grown in open field. **(E)** Auto-fluorescence of chloroplast of Pratap grown in open field (indicated by white arrow). **(F)** Merge image of **(D,E)**. Image size **(A–C)** 10 μm and **(D–F)** 25 μm.

In contrast, Pratap plants grown *in vitro* showed no bacterial signatures, although S9 stained the nuclei of cells, suggesting the absence of endophytic bacterial colonization under sterile conditions. Interestingly, bacterial signatures were detected in the leaves of Pratap plants grown in open field conditions, after staining with S9 (Figure 5). This observation suggests that environmental factors may influence bacterial colonization in Pratap plants.

### 3.5 Relative and absolute abundance of endophytic communities

Both Pratap and Podali Plants were grown in completely sterile condition in MS medium. At the phylum level, both Pratap and Podali plants were dominated by *Proteobacteria*, with Podali exhibiting 87.69% and Pratap 98.42% of the total bacterial community. However, a more detailed analysis at the genus level revealed significant differences between the two plant species.

In Pratap, a total of 10 Family were identified, with *Rhizobiaceae* being the most prominent. However, this family was present in only one of the replicates, and no bacterial family was consistently found across all three replicates, indicating a high degree of variability in the microbiome across replicates. Each Pratap replicate consisted of 20 plants.

**Figure 6:**
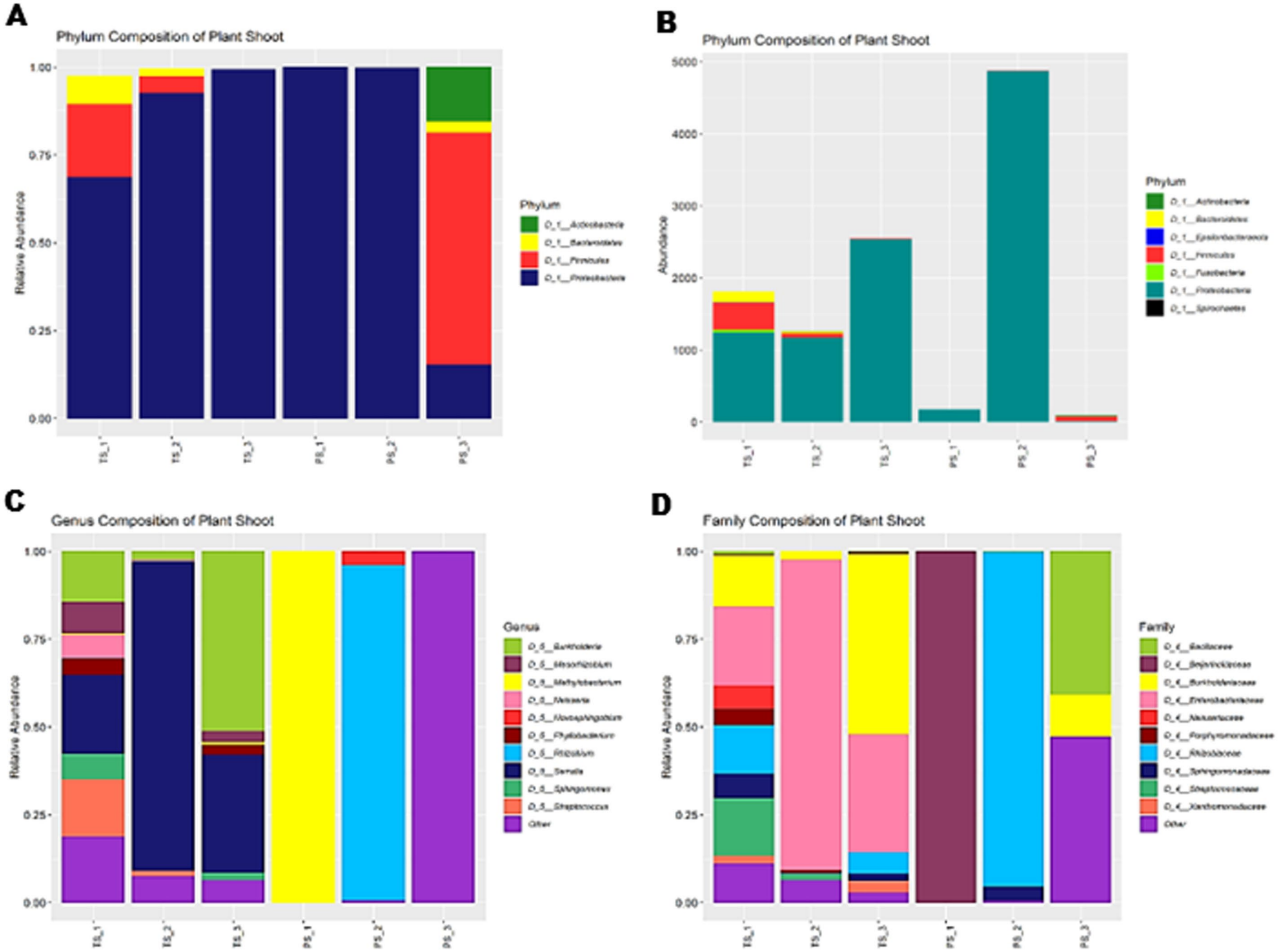
Diversity of bacterial endophyte In Tobacco Plant (TS) and Green Gram plants (PS). **(A)** Relative abundance at Phylum level. **(B)** Represent the absolute abundance of 3 replicates of tobacco var. Podali and Green Gram var. Pratap. **(C)** Relative abundance at genus level. **(D)** Relative abundance at family level.

In contrast, Podali displayed greater microbial diversity, with 37 families and 46 genera identified. *Burkholderiaceae*, *Beijerinckiaceae*, and *Rhizobiaceae* were present across all replicates of Podali, where each replicate consisted of a single plant. *Rhizobiales* were dominant in Pratap (93.8%), whereas in Podali, *Enterobacteriales* were more abundant, followed by *Betaproteobacteriales*. Additionally, seven genera were common between both plant species, but the remaining genera were unique to either Pratap or Podali. These results demonstrate a marked difference in bacterial load and community composition between the two plant species, with Podali harboring greater microbial diversity compared to Pratap (Figure 6).

## 4 Discussion

This study highlights several key findings regarding the optimization of callus induction, intracellular bacterial localization and the comparative analysis of endophytic communities in two plant species, Podali and Pratap. These results provide valuable insights into plant-microbe interactions under controlled versus natural conditions.

Callus induction in Podali was successfully optimized using exogenous auxins and cytokinins. Phenolic compound release from leaves was associated with tissue necrosis, whereas root explants formed friable green callus under light, indicating chlorophyll development. Light influenced callus morphology but was not essential for callus induction. Friable callus formed under light was more conducive for embryogenic cell suspension culture (Kong et al., 2023; Shin and Seo, 2018; Permadi et al., 2024). Similar intracellular associations have been documented in fungi, such as Candidatus *G. gigasporarum* in arbuscular mycorrhizal fungi (Lastovetsky et al., 2018), and endobacteria in Rhizopus leading to toxin production and reproductive changes (Partida-Martinez and Hertweck, 2005; Deehan et al., 2024). Recent work by Moebius et al. (2014), Richter et al. (2024), and Giger et al. (2024) further confirm bacterial colonization and manipulation of fungal host function. Our microscopy observations in Podali similarly support functional intracellular colonization. Young et al. (2025) expanded this concept to non-vascular plants, reporting endobacteria within liverwort oil bodies.

Transmission electron microscopy studies have long reported bacterial localization in plant cytoplasm and vacuoles (de Almeida et al., 2009). Verma et al. (2021) further supported the movement of EB from root to aerial tissue. Kim et al. (2015) highlighted the role of NCR211 peptides in intracellular bacterial survival in legumes. Live endophytic bacteria have been previously visualized with S9 (Thomas and Franco, 2021), including nuclear and cytoplasmic localization (Thomas and Reddy, 2013). While S9 can binds to the nucleic acids, its specificity against mitochondrial and chloroplast DNA remains underexplored. Our results demonstrate S9 specificity for bacterial and nuclear DNA, with no staining of chloroplasts. Co-staining with PI and MDR enables distinction between viable/non-viable cells and mitochondria. Bacteria and mitochondria were co-localized in inter-vacuolar strands (IVSs), suggesting IVSs facilitate bacterial movement (Gestel et al., 2002; Ruthardt et al., 2005; Madina et al., 2019). Co-staining with PI and MDR helped differentiate bacterial DNA from non-viable cells and mitochondria, respectively. Figure 2 shows bacterial co-localization with mitochondria in inter-vacuolar strands (IVSs) (Supplementary Figure S2; Supplementary Video V7). Time-lapse imaging confirmed bacterial motility through IVSs and cytoplasm. The observed co-localization of bacteria and mitochondria within IVSs suggests that bacteria, with their similar size range of 0.85–1 μm, can also utilize these channels for intracellular movement. Time-lapse imaging captured the movement of bacteria within IVSs and the surrounding cytoplasm (Supplementary Videos V1, V2). Suspension cultures grown in the dark lacked chlorophyll, resulting in no autofluorescence, while mitochondrial autofluorescence was seen in tissues. In Podali leaves, bacteria were observed near the nucleus and periphery. Pratap tissues grown *in vitro* showed no EB, despite successful nuclear staining, possibly due to low bacterial load or S9 limitations (McGoverin et al., 2020). Environmental exposure plays a significant role in colonization, as EB were seen in field-grown Pratap but not *in vitro*-grown plants (Akinde et al., 2016; Compant et al., 2019; Zhou et al., 2024).

NGS supported microscopy data, revealing predominance of *Proteobacteria* in both plants. Podali showed additional diversity *firmicutes* (7.89%) and *bacteroidetes* (3.08%). Pratap was largely dominated by *Proteobacteria* (98.42%) (*Firmicutes* 1.24%, *Actinobacteria* 0.27%). At genus and family levels, Podali hosted 46 genera and 37 families, while Pratap hosted 11 genera and 10 families. Only 5 genera and 7 families were shared between the two. Mann–Whitney U tests showed no statistically significant differences. PERMANOVA was unsuitable due to lack of overlap. These findings suggest distinct microbiomes influenced by genotype and cultivation. Podali replicates were derived from individual plants, yielding consistent profiles, whereas pooled Pratap samples may have diluted specific signals. This limits the use of statistical methods such as Spearman correlation or LEfSe. Similarly, ordination methods like PCA or PCoA were not applied due to distinct microbiome profiles. Thus, the study emphasizes presence/absence and descriptive analyses.

Low bacterial load in Pratap CLSM may account for reduced visibility. NGS is more sensitive to low-biomass detection than microscopy. Cancer cell lines and muscle tissues served as negative controls. In MCF-7 cells, S9 selectively stained nuclei; MDR labeled mitochondria (Supplementary Videos V3, V4). No chloroplast or mitochondria staining by S9 was observed, despite membrane similarities. Likely factors include differences in DNA accessibility or dye affinity. Our findings confirm EB presence in Podali cytoplasm, around nucleus, and membrane periphery. No EB were seen in *in vitro* Pratap but were found in field-grown samples (Supplementary Video V8), showing environment’s role. S9 limitations include inability to distinguish live vs. dead bacteria. Motility alone may not confirm viability, especially in streaming cytoplasm.

In summary, intracellular EB were observed in Podali cell suspensions and tissues, and in field-grown Pratap. S9 and MDR provided reliable staining, with no cross-reactivity. Illumina sequencing validated lower microbial load in Pratap. Findings underscore tissue culture’s influence on microbiome, limitations of current stains, and importance of environmental exposure. Further work is needed to determine EB function and host benefit under natural conditions.

## Data Availability

Data is available at this link: https://www.ncbi.nlm.nih.gov/sra/PRJNA1256188.

## Footnotes

^1^ https://assets.thermofisher.com/TFS-Assets/LSG/manuals/mp07007.pdf
