# Supplementary material for: Comparative analysis of endophytic bacterial localization and microbiome diversity in plant varieties under varied growth conditions through microscopic imaging and sequencing techniques
Source: Front Microbiol. 2025 May 16;16:1568209. doi: 10.3389/fmicb.2025.1568209 (PMC12122750; doi:10.3389/fmicb.2025.1568209)
Supplement: Supplementary file 1 [file Table_1.docx]

**Supplementary Information**

**Figure S1 - Tobacco var. Podali Seed Surface Sterilization and SEM Observation**. (A) Various bacteria are visible on the surface of unsterilized Tobacco var. Podali seeds. (B): No bacterial structures are observed following seed surface sterilization.

**Figure S2- Endophytic bacteria in Cytoplasm, Inter-vacuolar strand and around nucleus. (**A) Bacterial was observed in the cytoplasm, Inter vacuolar strand and around the nucleus. (B) Bright field image of the cell shows clear nucleus and Trans vacuolar strand. (C) merge image of A and B.

**Video V1- Bacterial tracking and cytoplasmic streaming in Plant cell suspension.** (A) Bacterial Tracking (BT) was observed around the nucleus and in cytoplasm’s shown in blue arrow B) Cytoplasmic streaming observed in Phase contrast image. C) Merge image of A and B.

**Video V2- Cytoplasmic streaming-** Under bright field microscope cytoplasmic streaming was observed around the cell periphery and in Inter-vacuolar strand as indicated by blue arrow.

**Video-V3. Observation of MCF-7 human cancer cell line under CLM.** A) Green fluorescence showed only nucleus, single cell was stain with S9 dye and does not stain other organelles. B) mitochondria of cancer cells were stained by MDR and shown in magenta color. C) merge image of A and B, Z-stacking step size 2-3µm.

**Video V4. distinction of chloroplast and nucleus with staining of S9 and MDR in Cardiac muscles.** 3D- Construct of animal Cardiac muscles showed that S9 only stains the nucleus as shown in green color. MDR stains only mitochondria as shown in magenta color. No bacterial and other organelle was observed. Z-stacking step size 2-3µm.

**Video V5- EB in the leaf tissue of tobacco plants.** Showed the bacteria around cell periphery indicated by green color and mitochondria in the cytoplasm indicated by magenta color.

**Video V6- of tobacco leaf tissue**. 3-D video of Podali plant tissue section- Endophytic bacteria showed around the nucleus and in cytoplasm, shown by fluorescence in green color. In magenta color showed the autofluorescence of chloroplasts. Z-Stacking step size 2-3µm.

**Video V7- Distinction of endophytic bacteria and chloroplast in tobacco plant cell suspension**. EB and chloroplasts are present in the Inter-vacuolar strand (IVS). EB are showed in green color and mitochondria in magenta.

**Video V8- EB in the shoot of GG plans growing in open field.** EB showed in green color, are observed in green gram plants shoot grown in open field. Autofluorescence of chloroplasts was observed in magenta color.
