# Supplementary figures and images for: Comparative analysis of endophytic bacterial localization and microbiome diversity in plant varieties under varied growth conditions through microscopic imaging and sequencing techniques
Source: Front Microbiol. 2025 May 16;16:1568209. doi: 10.3389/fmicb.2025.1568209 (PMC12122750; doi:10.3389/fmicb.2025.1568209)

## Slide 1
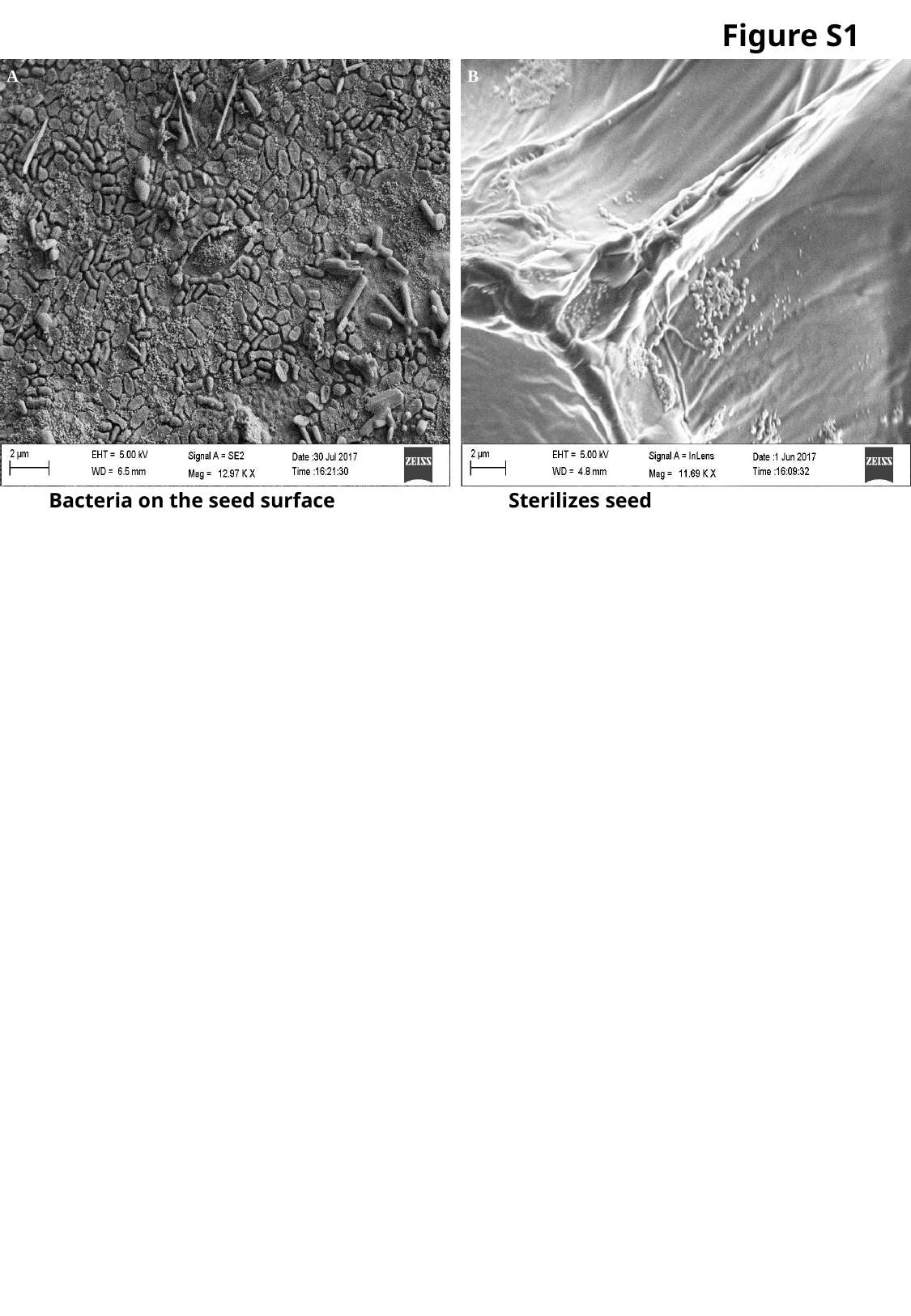

Figure S1
B
A
 Bacteria on the seed surface Sterilizes seed

Supplement: Supplementary file 2 [file Presentation_1.PPTX]

## Slide 1
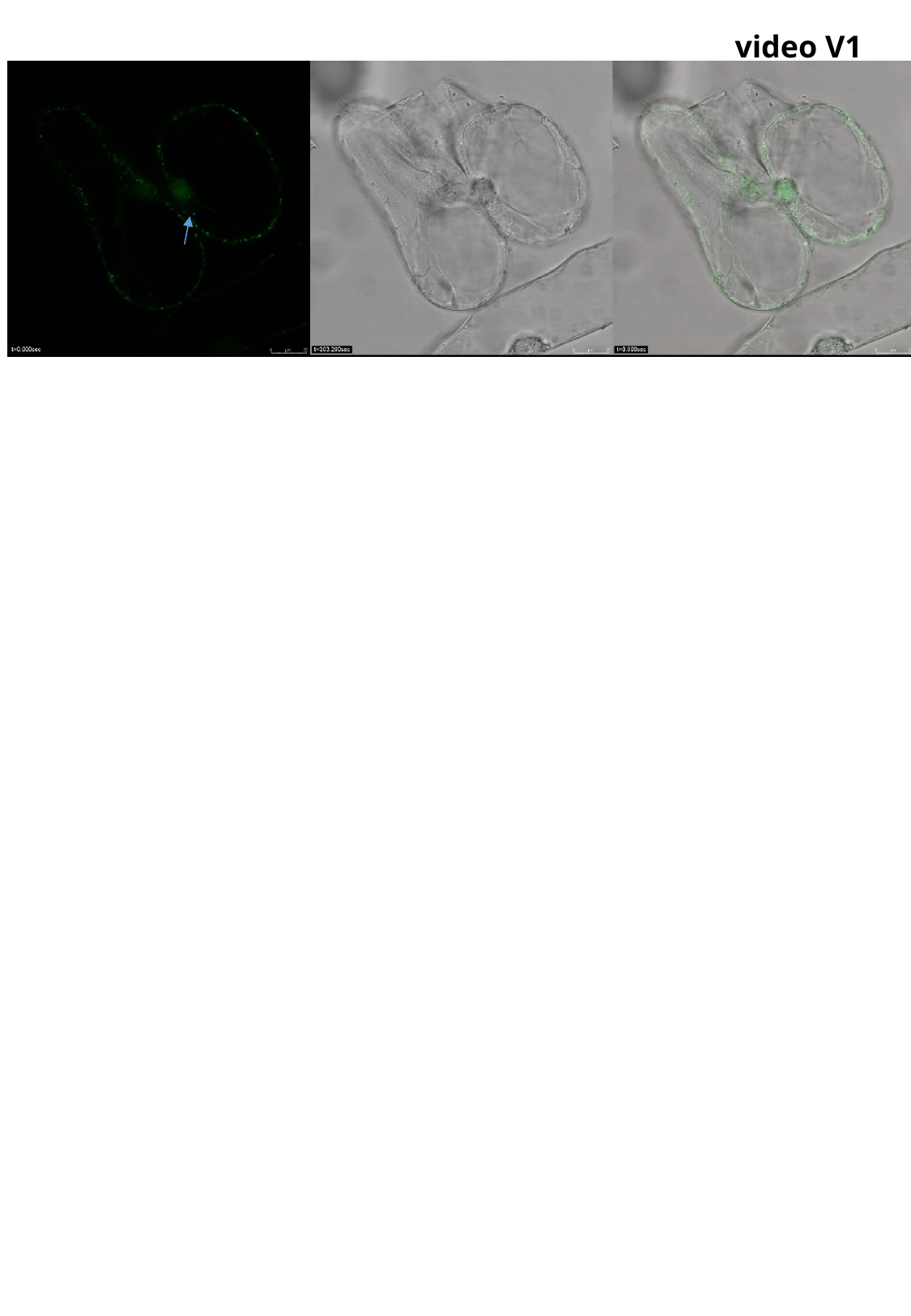

video V1
C
A
B
BT

Supplement: Supplementary file 4 [file Presentation_3.PPTX]

## Slide 1
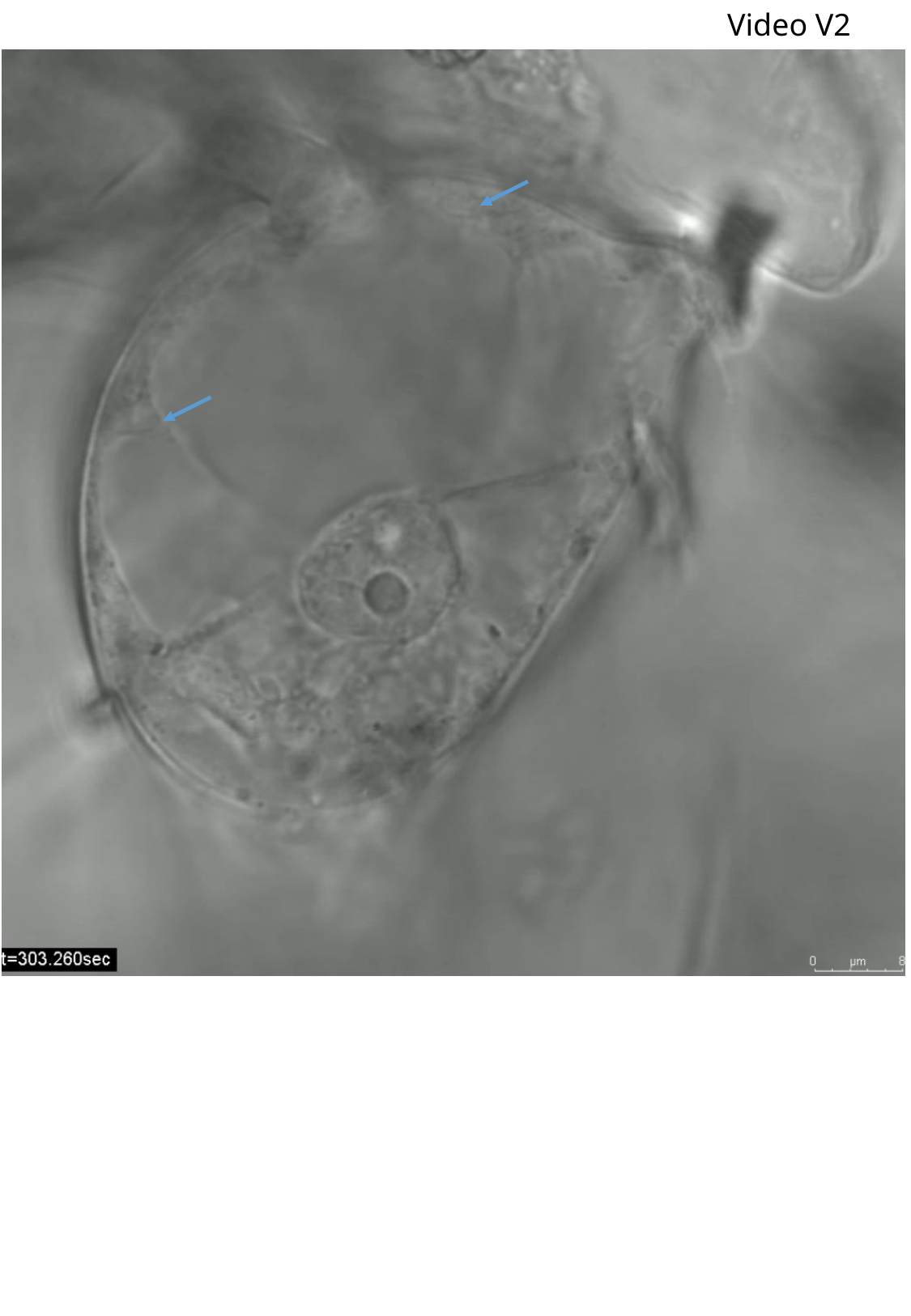

Video V2

Supplement: Supplementary file 5 [file Presentation_4.PPTX]

## Slide 1
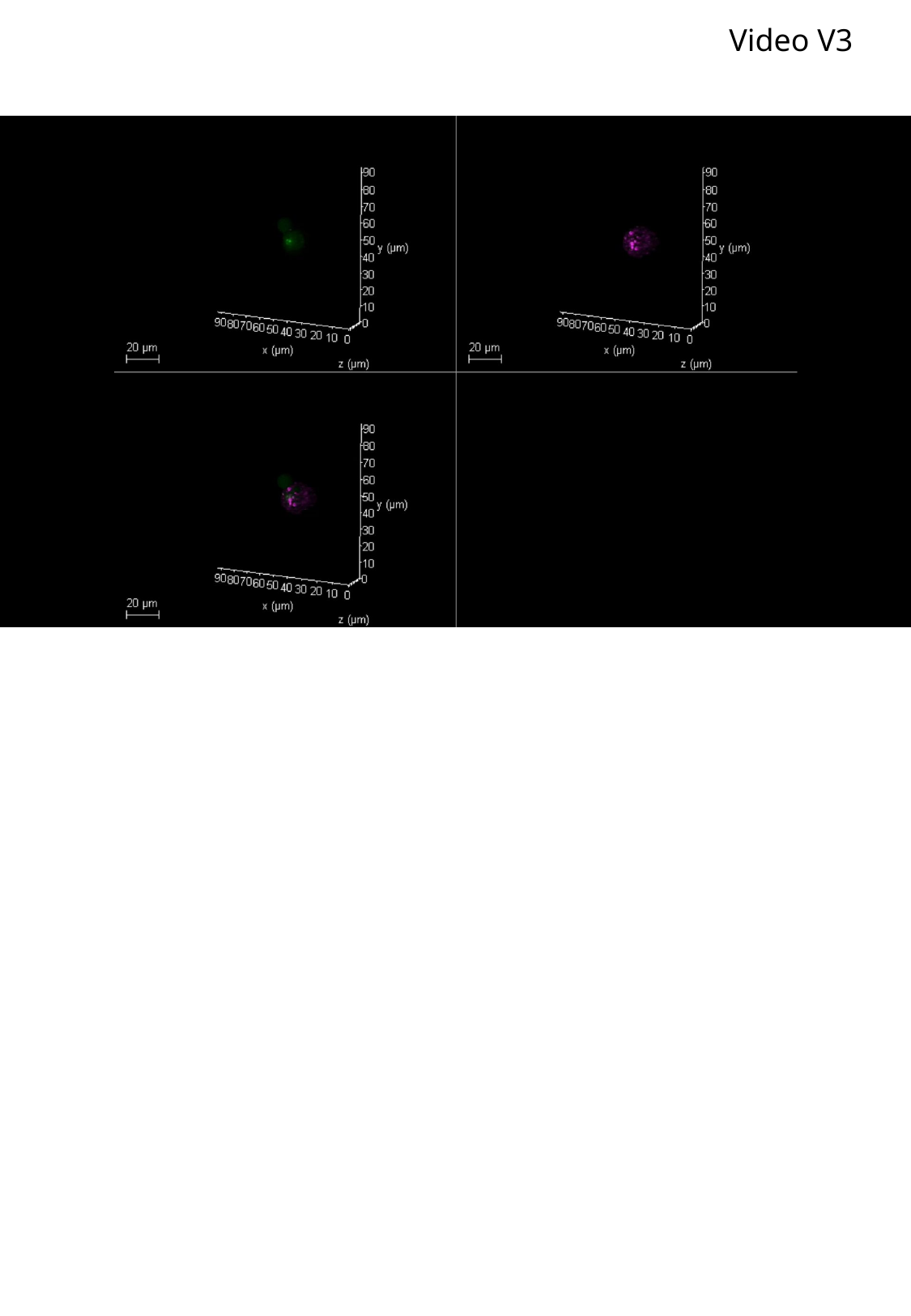

Video V3

Supplement: Supplementary file 6 [file Presentation_5.PPTX]

## Slide 1
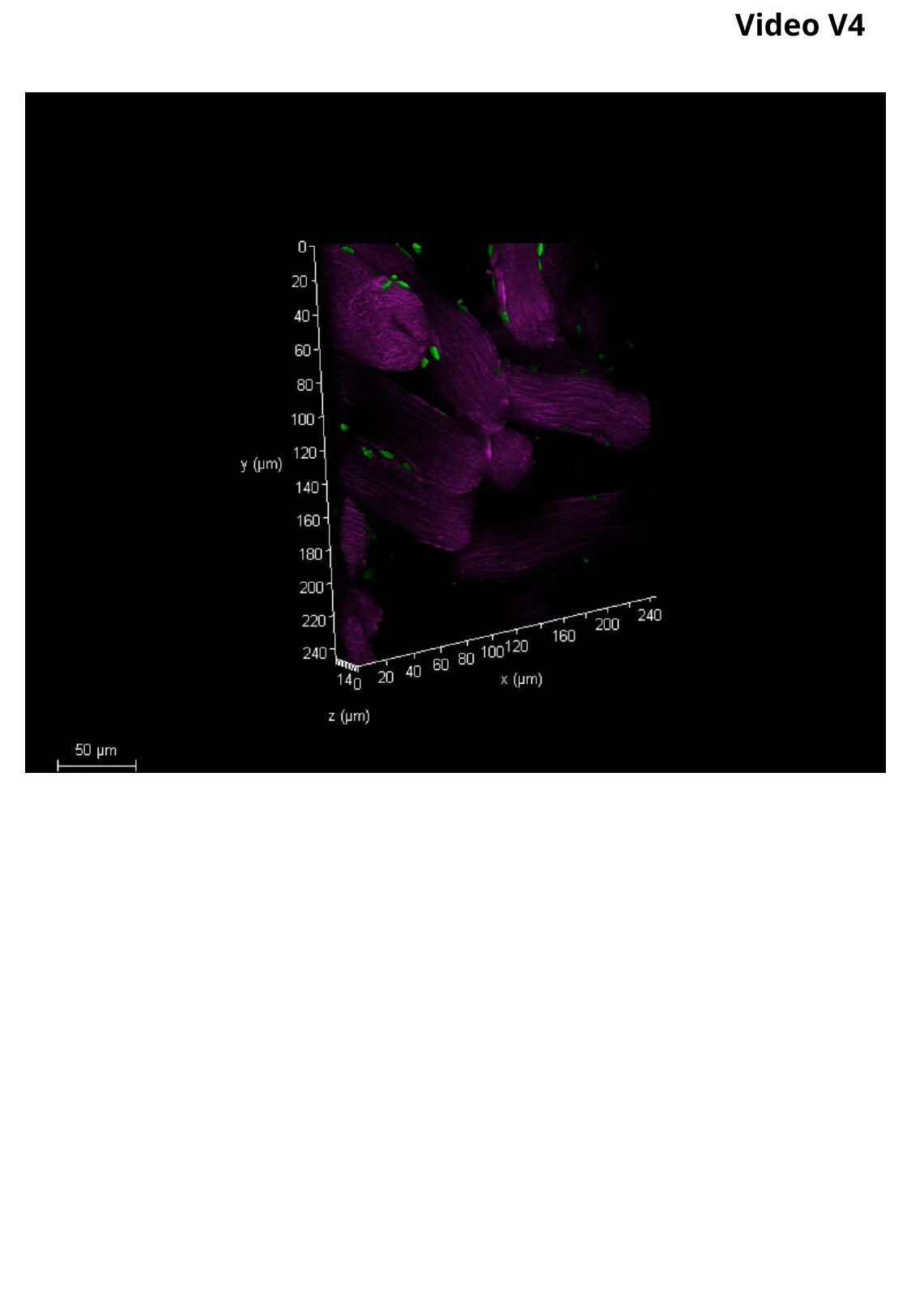

Video V4

Supplement: Supplementary file 7 [file Presentation_6.PPTX]

## Slide 1
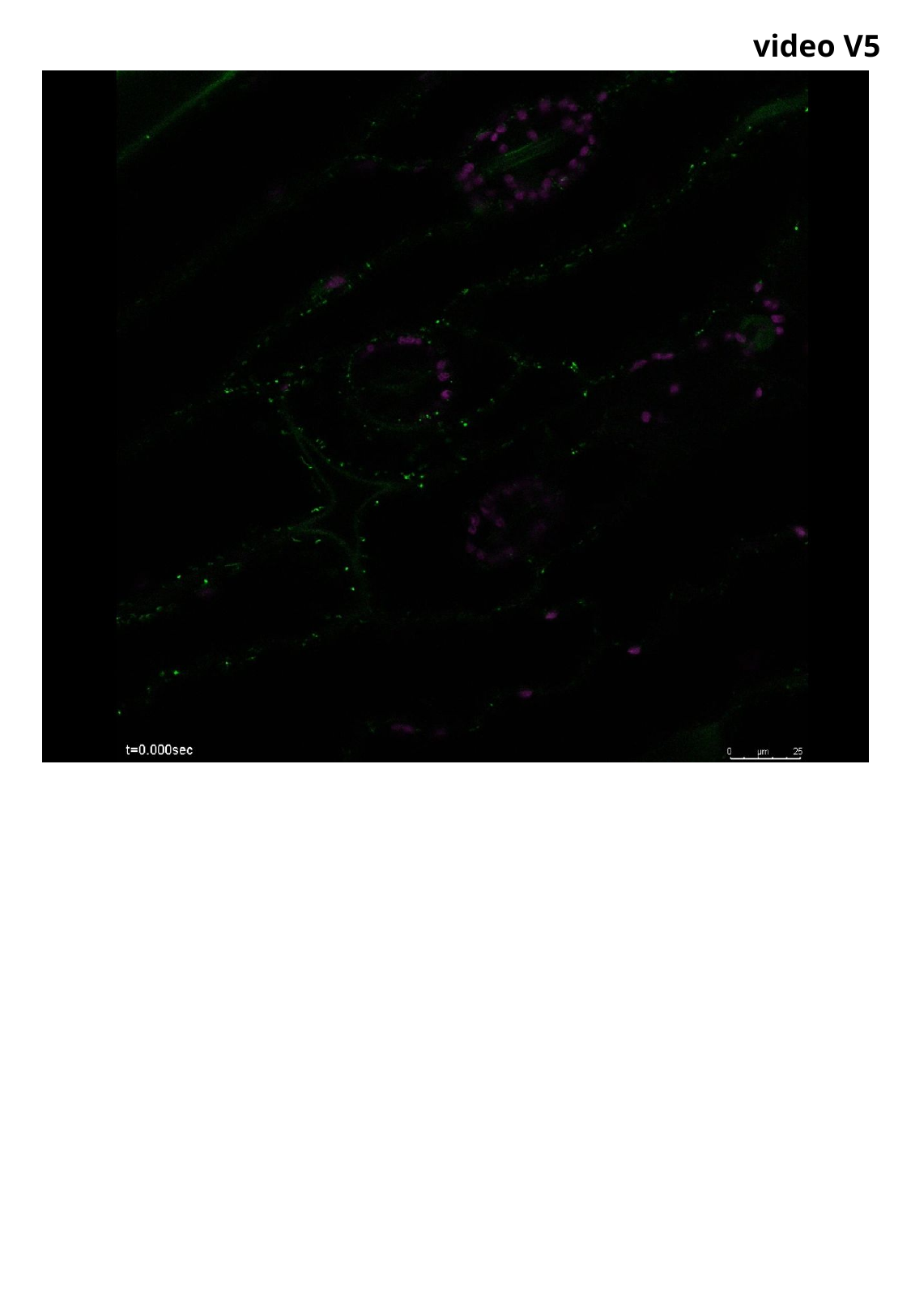

video V5

Supplement: Supplementary file 8 [file Presentation_7.PPTX]

## Slide 1
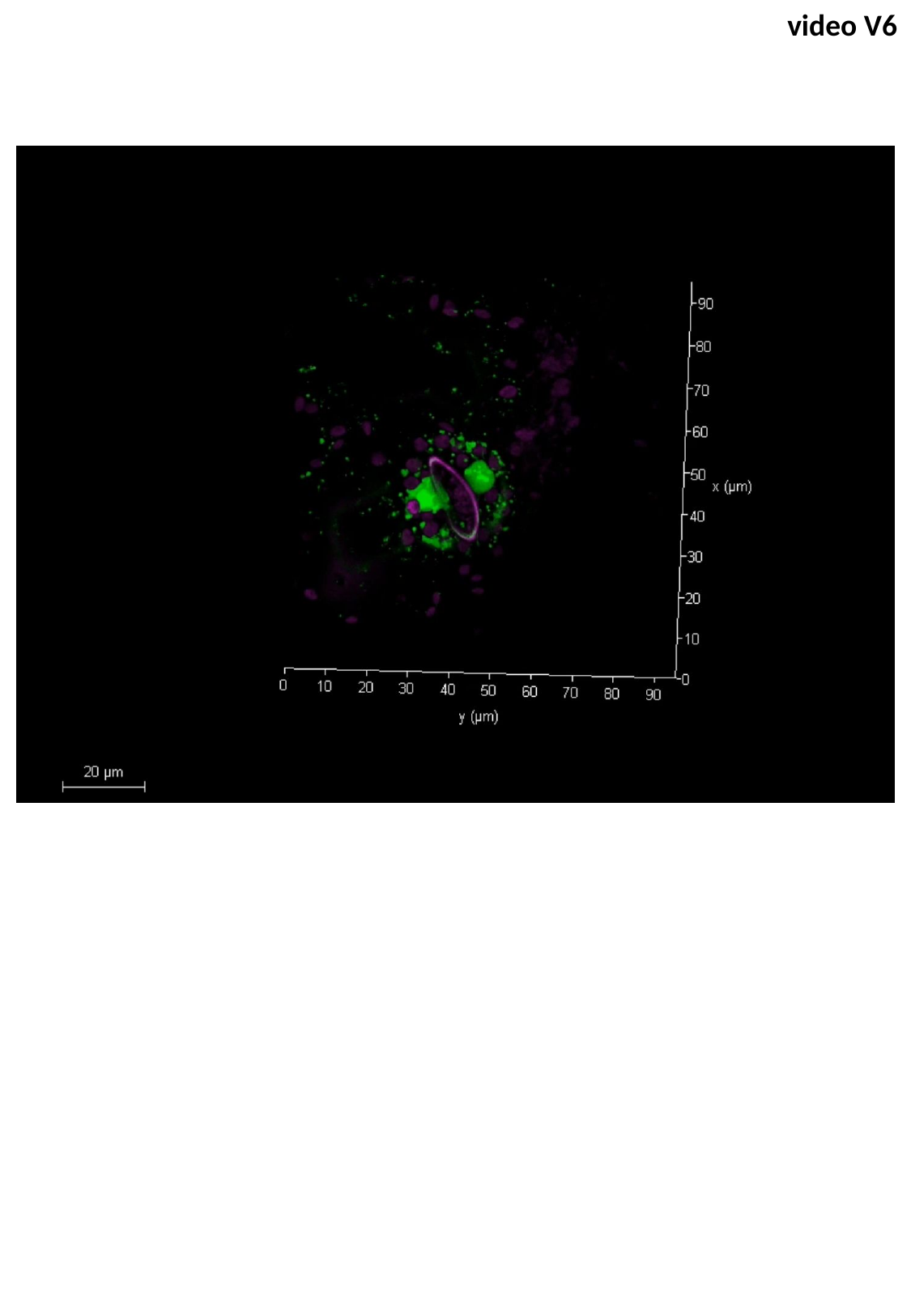

video V6

Supplement: Supplementary file 9 [file Presentation_8.PPTX]

## Slide 1
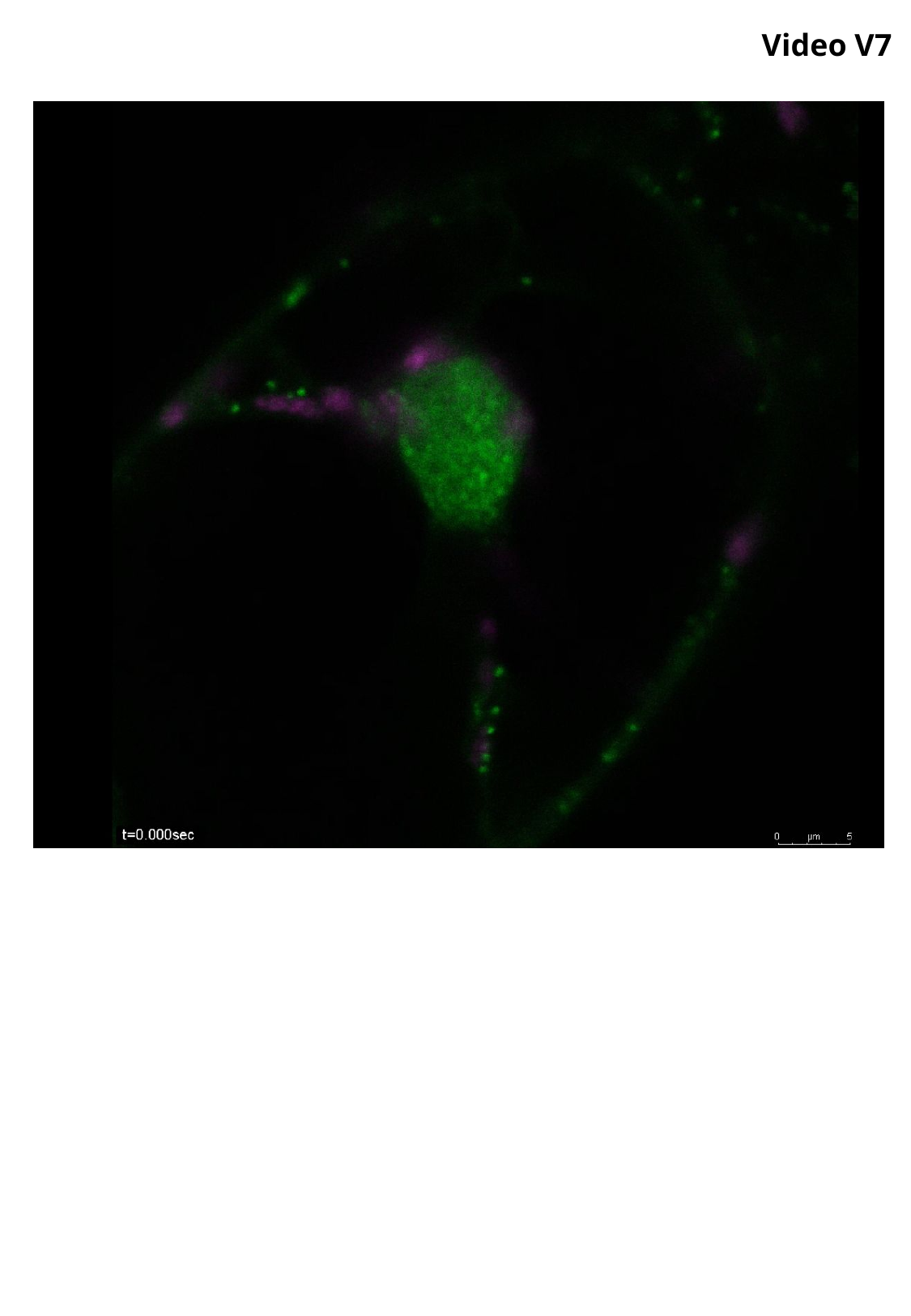

Video V7

Supplement: Supplementary file 10 [file Presentation_9.PPTX]

## Slide 1
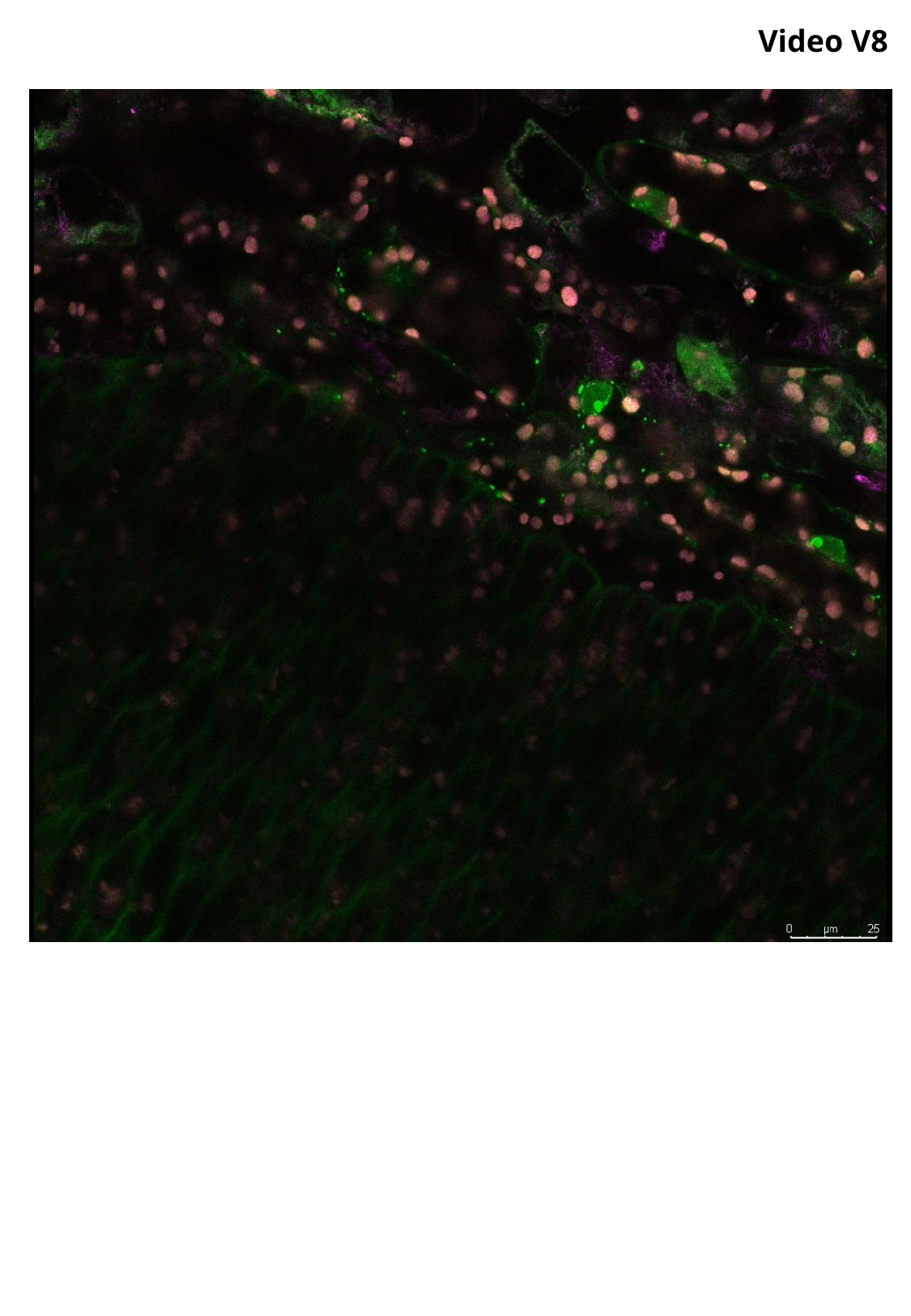

Video V8

Supplement: Supplementary file 11 [file Presentation_10.PPTX]
