# Supplementary material for: Comparative analysis of endophytic bacterial localization and microbiome diversity in plant varieties under varied growth conditions through microscopic imaging and sequencing techniques
Source: Front Microbiol. 2025 May 16;16:1568209. doi: 10.3389/fmicb.2025.1568209 (PMC12122750; doi:10.3389/fmicb.2025.1568209)
Supplement: Supplementary file 3 [file Presentation_2.PPTX]

## Slide 1
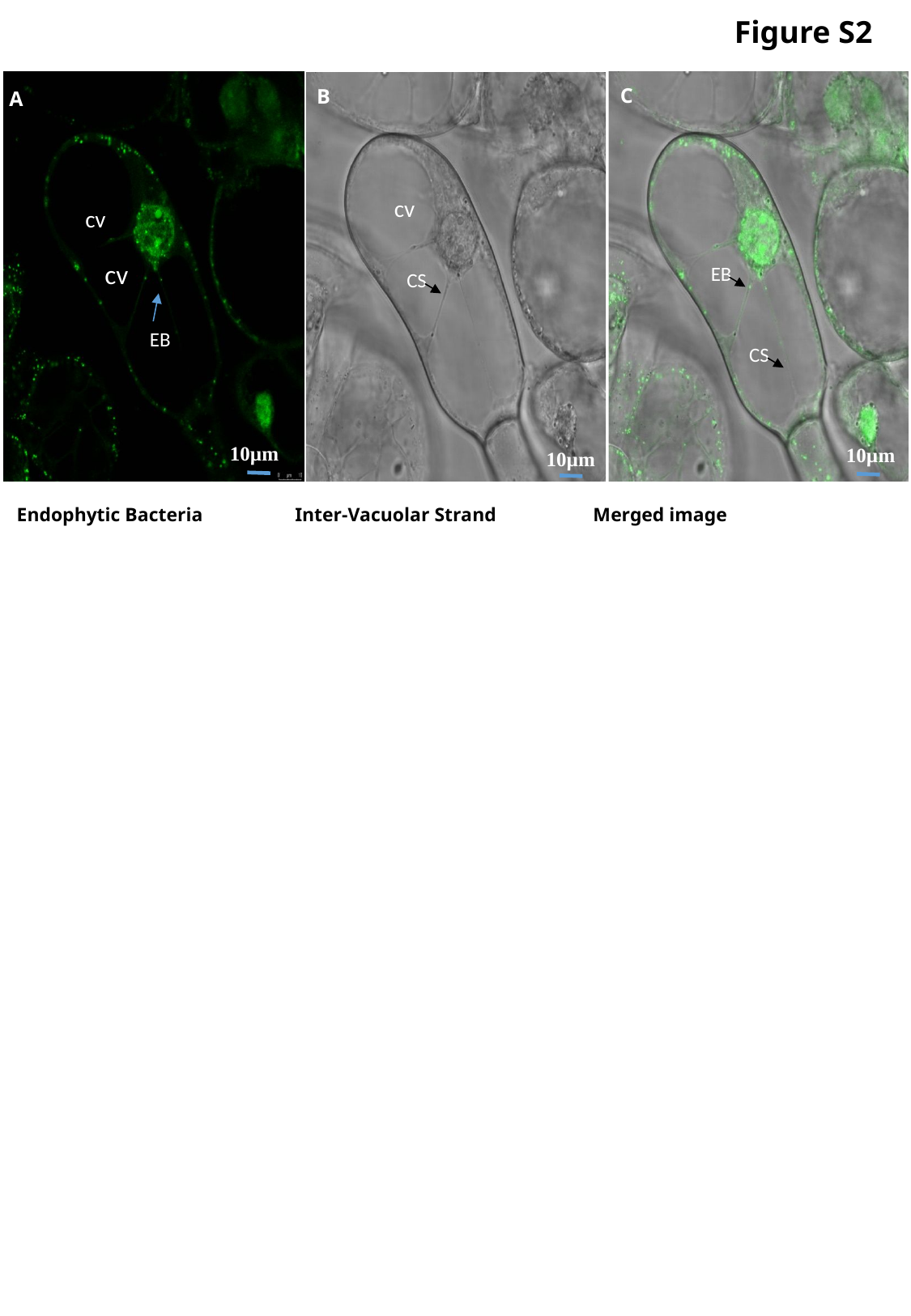

Figure S2
cv
cv
cv
EB
CS
EB
CS
10µm
10µm
10µm
C
B
A
 Endophytic Bacteria Inter-Vacuolar Strand Merged image
